# Psychosocial differences in perceived older workers’ work (un)adaptability, effectiveness and workplace age discrimination

**DOI:** 10.1192/j.eurpsy.2023.547

**Published:** 2023-07-19

**Authors:** S. von Humboldt, I. Miguel, J. Valentim, A. Costa, G. Low, I. Leal

**Affiliations:** ^1^William James Center for Research, ISPA - Instituto Universitário, Lisbon; ^2^Portucalense Institute of Human Development & Department of Psychology and Education, Portucalense University, Porto; ^3^Faculty of Psychology and Educational Sciences, University of Coimbra, Coimbra; ^4^ISPA - Instituto Universitário, Lisbon, Portugal; ^5^Faculty of Nursing, University of Alberta, Edmonton, AB, Canada

## Abstract

**Introduction:**

The aging population in the developed world has implied increasing age diversity in the workforce of organizations. Consequently, mutual perceptions about one’s co-workers and age discrimination is becoming increasingly important.

**Objectives:**

This study aims to explore how perceptions about older workers’ work (un)adaptability, work effectiveness and workplace age discrimination vary according to participants’ psychosocial factors, such as age group, gender, education level and work sector.

**Methods:**

This study included a sample of 453 workers in Portugal, diverse in terms of age, gender, education level and work sector. Four different instruments were used: (a) a sociodemographic questionnaire; (b) an older workers’ Work Adaptability scale; (c) an older workers’ Work Effectiveness Scale and; (d) the Workplace Age Discrimination Scale (WADS).

**Results:**

Middle-aged and older participants perceive older workers as more adaptable than younger participants. The oldest group of participants perceives older workers to be more work-effective and experience the highest levels of age discrimination in the workplace, when compared to the other age groups. Also, participants with lower levels of education tend to perceive higher levels of workplace age discrimination, when compared to participants with high school and higher education.

**Conclusions:**

Generational perceptions in the workplace are perceived by workers differently, hence organizations should implement age management strategies to address age discrimination, particularly due to the increasing proportion of older workers.

**Disclosure of Interest:**

None Declared

